# Patient risk evaluation for transcatheter aortic valve replacement (PRE-TAVR) — identification of real-time predictors of short- and long-term mortality

**DOI:** 10.1007/s00392-025-02704-6

**Published:** 2025-07-07

**Authors:** Julian Kreutz, Philipp Lauten, Georgios Chatzis, Marie Nabrotzki, Nikolaos Patsalis, Styliani Syntila, Harald Lapp, Bernhard Schieffer, Birgit Markus

**Affiliations:** 1 Department of Cardiology, Angiology, and Intensive Care Medicine, Philipps-Universität Marburg, Baldingerstraße, 35043 Marburg, Germany; 2 Department of Cardiology, Central Hospital of Bad Berka, Robert-Koch Allee 9, 99438 Bad Berka, Germany

**Keywords:** Transcatheter aortic valve replacement (TAVR), Predictors of mortality, 30-day mortality, 1-year mortality, LASSO regression analysis

## Abstract

**Background:**

The steadily increasing number of transcatheter aortic valve replacement (TAVR) procedures being performed on a heterogeneous patient population highlights the need for robust risk assessment. While EuroSCORE II is well established for surgical risks, it is less effective for TAVR, and the newer STS/ACC TAVR score has so far been validated mainly for in-hospital and 30-day mortality.

**Aims:**

This study aims to improve risk stratification for TAVR patients by identifying real-time predictors of 30-day and 1-year mortality that incorporate comprehensive, procedure-specific factors.

**Methods:**

Five-year data from 2256 transfemoral TAVR procedures performed at two German Heart Centers (2017–2022) were retrospectively analyzed. Predictors of 1-year and 30-day mortality were assessed using multivariable logistic and LASSO regression, considering a broad spectrum of patient demographics, comorbidities, and peri-procedural factors.

**Results:**

The analyses revealed a predictor model (PRE-TAVR predictors) for 1-year mortality (AUC 0.770; 95% CI 0.731–0.809), including age (> 81.5 years), NYHA stage IV, COPD (GOLD ≥ 2), atrial fibrillation, previous stroke or malignancy, elevated C-reactive protein (≥ 9.5 mg/L), aortic valve ΔP mean ≥ 48.5 mmHg, peripheral arterial disease (> stage 2) and low platelet count (≤ 228.5 g/L). The accuracy of the model exceeded the EuroSCORE II (AUC 0.645; 95% CI 0.599–0.691) and the STS/ACC TAVR score (AUC 0.714; 95% CI 0.670–0.758). For 30-day mortality, NYHA class IV was the only significant predictor in the bivariate analyses. However, additional LASSO analyses identified pre-existing renal insufficiency (KDIGO stage ≥ 3) and pre-TAVR sodium levels as further significant predictors. The AUC was 0.699 (95% CI 0.611–0.788) compared to an AUC of 0.680 (95% CI 0.604–0.756) for EuroSCORE II and 0.7129 (95% CI 0.633–0.793) for the STS/ACC TAVR score.

**Conclusion:**

The PRE-TAVR study developed a robust model, particularly for predicting 1-year mortality. This model outperformed the EuroSCORE II and STS/ACC TAVR scores, despite requiring fewer variables. It provides a solid basis for future risk scores and enables more precise patient selection.

**Graphical Abstract:**

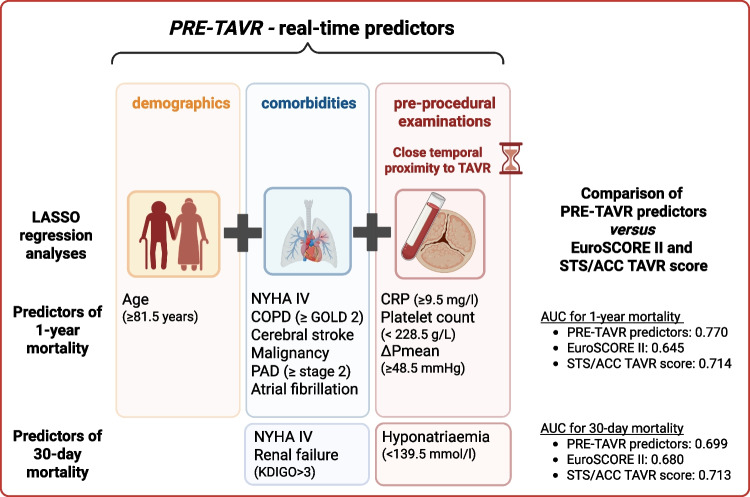

**Supplementary Information:**

The online version contains supplementary material available at 10.1007/s00392-025-02704-6.

## Introduction

In the past decades, transcatheter aortic valve replacement (TAVR) has become a well-established treatment option for patients with severe aortic stenosis. According to available study data, the therapy is currently applicable to a broad patient cohort. Initially limited to patients ineligible for or at high risk for surgical aortic valve replacement (SAVR), the indication has been extended in recent years to include those at intermediate and low risk, as well as patients at different stages and severity of the disease [1–6]. To optimize outcomes in these heterogeneous patient cohorts, this, in turn, underscores the need for assessing diverse and individualized risk profiles. Heart teams consistently rely on widely accepted surgical risk prediction models, such as the *European System for Cardiac Operative Risk Evaluation* (EuroSCORE II), to evaluate mortality and outcomes [7]. However, these models often fail to accurately predict mortality following interventional procedures such as TAVR, which significantly limits their validity and applicability [8, 9]. Although, over time, new TAVR-specific tools, such as the FRANCE-2 model, have shown superior performance compared to EuroSCORE II in external validation cohorts, their utility and clinical application remain limited [10, 11]. This limitation may stem from their static nature and broad temporal scope, which prevent them from capturing the dynamic range of relevant factors. The STS/ACC TAVR in-hospital mortality risk score, which uses 12 variables, has been evaluated for short-term outcomes and has demonstrated superior performance compared to existing models, establishing its potential as a valuable tool [12]. However, reliable predictors of 1-year mortality, which are essential for long-term outcomes, remain elusive. The aim of this study was to identify real-time predictors of both 30-day and 1-year mortality to improve the evaluation of the peri-interventional period and enable a more targeted approach to risk stratification. The term ‘real-time’ in this context, emphasizes the ability of these predictors to support clinical decision-making based on parameters obtained in close temporal proximity to the procedure, reflecting the patient’s actual clinical condition. Advanced statistical methods, including *Least Absolute Shrinkage and Selection Operator* (LASSO) analysis, were applied to improve predictive accuracy beyond traditional risk models. This approach seeks to facilitate more appropriate, timely, and personalized clinical decision-making.

## Materials and methods

### Study design

This multicenter study retrospectively analyzes comprehensive 5-year data (January 2017 and December 2022) from patients with severe aortic stenosis who underwent TAVR implantation at the Heart Centers of the University Hospital of Marburg and the Central Hospital of Bad Berka (Germany). Inclusion criteria for further data analysis were transfemoral TAVR implantation and availability of 30-day mortality data; no other exclusion criteria were applied, resulting in a consecutive cohort. To identify independent predictors of 30-day and 1-year mortality, this study compares data from TAVR survivors and non-survivors, focusing on the effects of demographics, comorbidities, and pre-interventional examinations, including electrocardiograms (ECG), chest X-rays, blood tests, computed tomography, and echocardiography. Additionally, it correlates peri-interventional complications with 30-day and 1-year mortality**.** The EuroSCORE II and the STS/ACC TAVR in-hospital mortality risk score were calculated using data from hospital records, but only for patients who had complete data for all the necessary variables.

Patient selection followed the *European Society of Cardiology* (ESC)/*European Association for Cardio-Thoracic Surgery* (EACTS) guidelines for TAVR implantation. Periprocedural management was similar following comparable standard operating procedures (SOPs) in both heart centers, with a consistent team of experienced professionals over the years. Patient cases were pre-evaluated and reviewed by each center’s heart team. Data collection included physician letters, laboratory results, admission ECGs, imaging reports, and procedural protocols.

### Statistical analysis

#### Descriptive statistics and bivariate analyses

Statistical analyses were performed using SPSS (IBM, version 29) and Stata (StataCorp LLC., version 16) to compare data between 30-day TAVR survivors and non-survivors as well as between 1-year TAVR survivors and non-survivors. Continuous variables were assessed for normality and described as mean ± standard deviation or median and interquartile range (IQR) for non-normal distributions. Categorical variables were summarized using counts and percentages. Bivariate analyses were conducted to compare TAVR survivors and non-survivors using *t*-tests or Mann–Whitney tests for continuous variables and Chi-squared tests or Fisher’s exact tests for categorical variables, with a significance level set at *p* < 0.05.

#### Handling of missing data

Missing data was handled using the multiple imputation procedure in Stata 16.1, including all variables intended for subsequent testing. Twenty imputations were generated for each missing value. The imputation model used linear regression for continuous variables and logistic regression for categorical variables, maintaining consistency across variable types.

#### LASSO regression analyses

Additionally, to identify predictors of 1-year and 30-day mortality, LASSO regression analyses were conducted. LASSO regression is effective at refining models by shrinking irrelevant coefficients toward zero and selecting the most informative predictors. This approach reduces the risk of overfitting and stabilizes estimates when predictors are highly correlated, or multicollinear. It is also well-suited for high-dimensional datasets. To address the missing data, we performed multiple imputation and created 20 complete datasets. We then performed LASSO regression separately on each of these 20 imputed datasets, producing slightly different models depending on the imputed values. We considered variables that appeared in at least 10 of the 20 models to be robust or “stable” predictors. We then included these stable predictors in multivariable logistic regression analyses to identify independent predictors of 1-year and 30-day mortality.

#### Multivariable regression analyses and model performance assessment

Multivariable regression analyses were performed to identify independent predictors of 1-year and 30-day mortality from statistically significant factors in the previous bivariate comparisons. Additionally, multivariable regression analyses were conducted using the variables identified in the LASSO analyses. Stata software was used to calculate the area under the curve (AUC) of the receiver operating characteristic (ROC) for logistic regression models and compare them with EuroSCORE II predictions for patients for whom this information was available. To compare the differences between the AUC of the ROC curves, the DeLong test was employed. Relevant biomarkers were log-transformed for analysis, and further regression models calculated odds ratios (OR) and confidence intervals (CI) to the second decimal place. It should be noted that OR values of 1.00 were never included in the models but could be the result of rounding to the second decimal place. Cut-off values were determined using Youden’s index for each identified significant predictor.

#### Positive and negative predictive value calculations

To provide additional clinical context for our models, we calculated the positive and negative predictive values for 1-year and 30-day mortality. We used the actual survival outcome (alive or deceased) as the “true state” and the logistic regression model predictions as the “test result.” We performed logistic regressions to generate continuous linear predictor values (log-odds). To dichotomize these values, we identified the optimal cutoff using Youden’s index to maximize sensitivity and specificity for our dataset. Using these thresholds, we created binary predictions of “likely dead” or “likely alive.” From the resulting 2 × 2 tables, we calculated the PPV, NPV, sensitivity, and specificity (with 95% confidence intervals). We applied the same approach to the EuroSCORE II and STS/ACC TAVR score for direct comparison.

#### Correlation analysis of peri-interventional complications

Moreover, correlation analysis was employed to assess the relationship between peri-interventional complications and 30-day and 1-year mortality. The Phi coefficient for binary variables assessed the impact of specific complications on mortality. For continuous or ordinal variables, Pearson’s *r* quantified linear relationships. Values close to ± 0.1 indicated weak, ± 0.3 moderate, and ± 0.5 strong correlations, up to ± 1 for very strong correlations.

### Clinical trial registration and ethics approval

The study was registered in the German Clinical Trials Register (ID DRKS00035463) and approved by the local ethics committees of the Philipps University of Marburg (approval date: 27 October 2022) and the Ethics Committee of the Thuringia Medical Association (approval date: 13 July 2023) in accordance with the Declaration of Helsinki.

## Results

### Study cohort

**Fig. 1:**
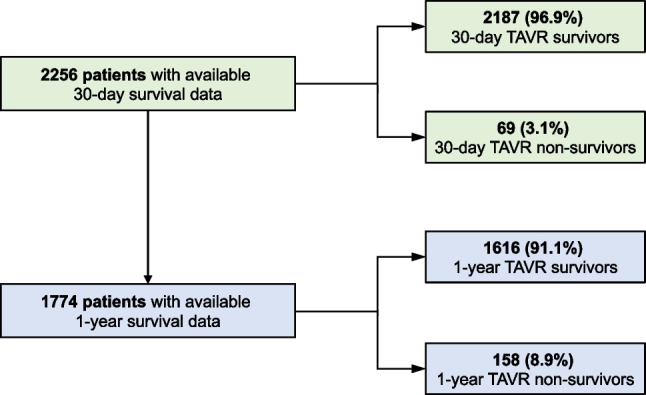
Study cohort

This retrospective analysis included data from 2256 patients (Fig. 1) who underwent transfemoral TAVR implantation at one of the two participating centers during the study period and for whom 30-day mortality data were available (5 patients were previously excluded due to missing data). 1-year survival data were available for 1774 patients. Of these patients, 1616 (91.1%) survived one year after TAVR implantation (1-year TAVR survivors) and 158 (8.9%) died within the first year (1-year TAVR non-survivors). The median EuroSCORE II score was 4.6 (IQR 2.7–8.5) for 1-year TAVR survivors and 7.0 (IQR 4.2–14.8) for 1-year TAVR non-survivors (*p* < 0.001). The first 30 days were survived by 2187 (96.9%) of the 2256 patients (30-day TAVR survivors), while 69 patients (3.1%) died within the first 30 days (30-day TAVR non-survivors). The overall outcome of the entire study cohort remained stable over the years without significant changes. The median EuroSCORE II score was 4.5 (IQR 2.7–8.1) for 30-day TAVR survivors and 6.9 (IQR 4.5–16.4) for 30-day TAVR non-survivors (*p* < 0.001). One thousand four hundred forty-nine patients received the Edwards Sapien 3 balloon-expanding system. In 587 patients, the Medtronic CoreValve/Evolut platform self-expanding system was implanted, and in 391 patients the Boston Scientific SYMETIS ACURATE neoTM TF self-expanding system. The Boston Scientific LotusEdge mechanical expansion system was implanted in 29 patients.

### Identification of predictors of 1-year mortality

To determine the predictors of 1-year mortality, the TAVR patient cohort was divided into two subgroups: 1-year TAVR survivors and non-survivors. First, demographic data and comorbidities of both subgroups were compared in a bivariate comparative analysis and LASSO analysis. Results, including *p*-values from bivariate comparisons and frequencies of LASSO models including each variable, are shown in Table 1.

**Table 1:** Demographics and comorbidities of 1-year TAVR survivors vs. non-survivors

	*n* =	1-year TAVR survivors	1-year TAVR non-survivors	*p*-value	Number of LASSO models
Number of patients^a^	1774	1616 (91.6)	158 (8.9)		
Age (years)^b^	1774	79.0 (± 6.4)	81.4 (± 5.8)	< 0.001	20
Male sex^a^	1774	852 (52.7)	95 (60.1)	0.075	20
BMI (kg/m^2^)^b^	1771	28.8 (± 5.4)	28.1 (± 4.9)	0.136	19
CHD ^a^	1774	872 (54.0)	102 (64.6)	0.011	20
Cardiac bypass surgery^a^	1773	96 (5.9)	12 (7.6)	0.408	0
Atrial fibrillation^a^	1774	538 (33.3)	84 (53.2)	< 0.001	20
PAD (≥ stage 2)^a^	1773	114 (7.1)	21 (13.3)	0.005	20
Heart failure ≥ NYHA III^a^	1745	1083 (68.2)	128 (82.1)	< 0.001	20
Arterial hypertension^a^	1773	1409 (87.2)	138 (87.3)	0.972	0
Diabetes mellitus^a^	1773	676 (41.9)	67 (42.4)	0.894	15
Insulin-dependent diabetes mellitus^a^	1725	276 (17.1)	25 (22.7)	0.131	0
Nicotine abuse (> 5py)^a^	1774	248 (15.3)	26 (16.5)	0.713	0
Chronic renal failure KDIGO ≥ stage 3^a^	1774	629 (38.9)	99 (62.7)	< 0.001	20
Need for renal replacement therapy^a^	1774	26 (1.6)	3 (1.9)	0.739	14
COPD ≥ GOLD 2^a^	1774	158 (9.8)	29 (18.4)	< 0.001	20
Cerebral Stroke^a^	1773	46 (2.8)	11 (7.0)	0.005	20
Malignant disease^a^	1773	90 (5.6)	19 (12.0)	0.001	20
Pacemaker^a^	1771	176 (10.9)	13 (8.3)	0.309	20

*n* number of patients with valid data, *BMI *body mass index, *CHD* coronary heart disease, *PAD* peripheral arterial disease, *NYHA* New York Heart Association, *py* pack years, *KDIGO* Kidney Disease Improve Global Outcomes, *COPD* chronic obstructive pulmonary disease

^a^n (%); ^b^Mean(SD)

Subsequently, bivariate comparisons and LASSO analysis were made for the pre-interventional examinations (ECG, chest x-ray, blood values in the 24 h before TAVR, computed tomography, and echocardiography) according to the pre-described statistical procedures (Table 2).

**Table 2:** Pre-interventional examinations of 1-year TAVR survivors versus non-survivors

	*n* =	1-year TAVR survivors	1-year TAVR non-survivors	*p*-value	Number of LASSO models
**Pre-interventional ECG**					
Heart rate at rest (bpm)^b^	1761	75.2 (± 14.2)	76.2 (± 15.9)	0.447	0
Right bundle branch block^a^	1774	138 (8.5)	16 (10.0)	0.499	0
Left bundle branch block^a^	1774	148 (9.2)	19 (12.0)	0.239	0
First degree AV block^a^	1774	176 (10.9)	11 (7.0)	0.125	20
**Pre-interventional chest X-ray**					
Pleural effusion^a^	1774	174 (10.8)	31 (19.6)	< 0.001	6
Other signs of congestion^a^	1774	87 (5.4)	15 (19.5)	0.034	0
**Pre-interventional blood values (24 h before TAVR)**					
CRP (mg/dL)^c^	1768	2.9 (1.2–7.3)	5.4 (2.2–14.0)	< 0.001	20
HbA1c (%)^b^	1724	6.2 (± 0.9)	6.2 (± 1.1)	0.800	0
Hemoglobin (g/L)^b^	1774	126.5 (± 17.4)	119.7 (± 19.9)	< 0.001	20
Hematocrit (L/L)^b^	1772	0.382 (± 0.047)	0.365 (± 0.054)	< 0.001	0
Leucocyte count (g/L)^c^	1766	7.7 (6.5–9.3)	8.1 (6.5–9.7)	0.187	0
Platelet count (g/L)^b^	1774	214.5 (± 69.2)	202.9 (± 80.7)	0.480	20
Sodium (mmol/L)^b^	1760	140.9 (± 3.4)	140.0 (± 4.9)	0.034	20
NT-proBNP (pg/mL)^c^	1267	1514.0 (579.0–3520.3)	3942.0 (1293.0–7774.5)	< 0.001	20
**Pre-interventional CT scan**					
Diameter of aortic valve annulus^b^	1746	24.6 (± 2.4)	24.8 (± 2.4)	0.536	0
Diameter of ascending aorta^b^	1209	34.8 (± 4.4)	34.8 (± 4.3)	0.931	11
Bicuspid aortic valve ^a^	1774	166 (10.3)	12 (7.6)	0.285	0
**Pre-interventional echocardiography**					
LVEF ≤ 45%^a^	1763	403 (25.1)	48 (30.6)	0.133	19
Vmax (mmHg)^b^	1543	4.2 (± 0.7)	4.0 (± 0.7)	0.002	0
ΔPmean (mmHg)^b^	1629	45.6 (± 15.6)	40.1 (± 15.3)	< 0.001	20
Vitium (grade 2/3) of the mitral valve^a^	1747	513 (32.2)	61 (39.1)	0.082	14
Vitium (grade 2/3) of the tricuspid valve^a^	1748	333 (20.9)	57 (36.5)	< 0.001	20

*n* number of patients with valid data, *bpm* beats per minute, *AV Block* atrioventricular block, *CRP* C-reactive protein, *HbA1c* hemoglobin A1c, *NT-proBNP* N-terminal prohormone of brain natriuretic peptide, *LVEF* left ventricular ejection fraction, *Vmax* peak transvalvular velocity of aortic valve, *ΔPmean* mean aortic valve pressure gradient

^a^n (%); ^b^Mean (SD); ^c^Median (IQR)

#### Multivariable regression analysis of parameters from bivariate comparisons between 1-year TAVR survivors and non-survivors

In a second step, multiple regression analysis (Supplementary Table 1) was performed to identify independent predictors of 1-year mortality using variables that previously showed significant differences between 1-year TAVR survivors and non-survivors (Tables 1 and 2). Since the analysis revealed a high correlation between hemoglobin and hematocrit, which led to large confidence intervals for hematocrit, the variable “hematocrit” was excluded from the subsequent models. NYHA III and NYHA IV stages were treated separately. Moreover, predictors with no significant *p*-values were progressively excluded to simplify the model. The final regression model (Table 3), which included 1410 patients with available EuroSCORE II data, achieved an AUC of 0.747 (95% CI 0.705–0.789). This model significantly outperformed both the EuroSCORE II (AUC 0.645, 95% CI 0.599–0.691) and the STS/ACC TAVR score (AUC 0.7139, 95% CI 0.670–0.758), as validated by the DeLong test (*p* < 0.001). Cut-off values of these predictors were established using the Youden index (Table 3).

**Table 3:** Predictors for 1-year mortality from multivariable regression analysis including significant variables from Supplementary Table 1 and cut-off values for predictors using the Youden index. Parameters were initially identified from the bivariate comparisons between 1-year TAVR survivors and non-survivors from Table 1 (demographics and comorbidities) and Table 2 (pre-interventional examinations). Predictors that were found to be non-informative were previously excluded

	OR (95% CI)	*p*-value	Cut-off value
Age (years)	1.07 (1.04–1.12)	< 0.001	81.5
Heart failure NYHA IV	1.97 (1.25–3.11)	0.004	/
COPD ≥ GOLD 2	2.27 (1.39–3.72)	0.001	/
Cerebral stroke	2.99 (1.35–6.65)	0.007	/
Malignant disease	2.44 (1.32–4.51)	0.005	/
Atrial fibrillation	1.71 (1.16–2.52)	0.007	/
CRP (mg/l)	1.22 (1.06–1.41)	0.005	9.5
ΔPmean (mmHg)	0.98 (0.97–1.00)	0.012	48.5

*NYHA* New York Heart Association, *COPD* chronic obstructive pulmonary disease, *CRP* C-reactive protein, *ΔPmean* mean aortic valve pressure gradient

#### LASSO analysis for identifying predictors of 1-year mortality

**Fig. 2:**
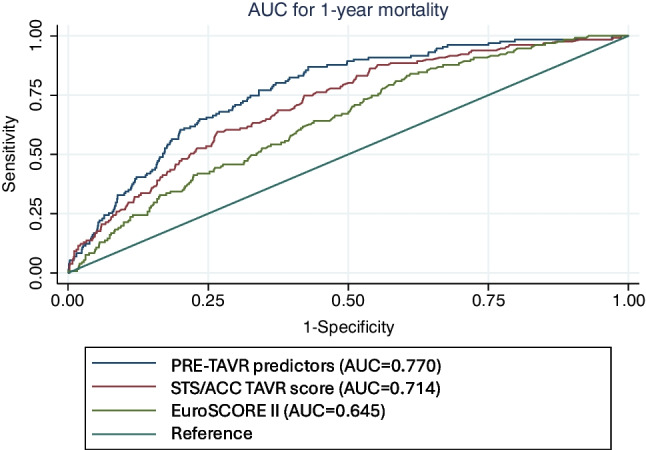
Comparative analysis of the predictive power of different models. ROC curves for survival comparing the *PRE-TAVR* predictor model in Table 4 (blue curve, AUC 0.770 [95% CI 00.731–0.809]) with the STS/ACC TAVR score (green curve, AUC 0.7139 [95% CI 0.66994–0.75791] and the EuroSCORE II (red curve, AUC 0.645 [95% CI 0.599–0.691])

For the LASSO analysis, variables included in at least 10 of 20 LASSO models from previous analyses (Tables 1 and 2) were identified as stable predictors and further evaluated in multiple regression analyses (Supplementary Table 2). NYHA stages III and IV were analyzed separately, analogous to the previous multiple regression model (“Multivariable regression analysis of parameters from bivariate comparisons between 1-year TAVR survivors and non-survivors” section). The original imputed model was confirmed by a robustness analysis with non-imputed data, which showed no relevant differences. Once again, predictors with a *p*-value > 0.05 were progressively excluded to simplify the model. In the LASSO analysis, the previously identified *PRE-TAVR* predictors were confirmed, and peripheral arterial disease (≥ stage 2) and the platelet count before TAVR were also shown to be significant predictors, with cut-off values identified using the Youden index (Table 4). The final predictor model for 1-year mortality (*PRE-TAVR* predictors), which included 1410 patients with fully documented data, had an AUC of 0.770 (95% CI 0.731–0.809). This significantly exceeded the AUCs of the EuroSCORE II (0.645, 95% CI 0.599–0.691) and the STS/ACC TAVR score (0.7139, 95% CI 0.66994–0.75791), and the superiority was confirmed by a DeLong test with a *p*-value of < 0.001. The corresponding ROC curves are shown in Fig. 2. Additionally, the PRE-TAVR model demonstrated a PPV of 17.2% (95% CI 14.4–20.3%) and a NPV of 97.7% (95% CI 96.4–98.7%). For comparison, the EuroSCORE II had a PPV of 12.3% (95% CI 10.3–14.7%) and a NPV of 96.0% (95% CI 93.9–97.5%). The STS/ACC TAVR score achieved a PPV of 18.6% (95% CI 15.0–22.7%) and an NPV of 94.7% (95% CI 93.1–96.0%).

**Table 4:** *PRE-TAVR* predictors (LASSO analysis). Multivariable regression analysis including significant variables from Supplementary Table 2 and cut-off values for predictors using the Youden index. Parameters were initially identified from the LASSO analyses between 1-year TAVR survivors and non-survivors from Table 1 (demographics and comorbidities) and Table 2 (pre-interventional examinations). Predictors that were found to be non-informative were previously excluded

	OR (95% CI)	*p*-value	Cut-off value
Age (years)	1.08 (1.04–1.12)	< 0.001	81.5
Heart failure NYHA IV	1.95 (1.23–3.09)	0.004	/
COPD ≥ GOLD 2	2.23 (1.38–3.73)	0.001	/
Cerebral stroke	3.05 (1.36–6.85)	0.007	/
Malignant disease	2.35 (1.27–4.38)	0.007	/
PAD (≥ stage 2)	2.22 (1.27–3.90)	0.005	/
Atrial fibrillation	1.71 (1.16–2.53)	0.007	/
CRP (mg/L)	1.25 (1.08–1.44)	0.002	9.5
Platelet count (g/L)	1.00 (0.99–1.00)	0.012	228.5
ΔPmean (mmHg)	0.98 (0.97–1.00)	0.016	48.5

*NYHA* New York Heart Association, *COPD* chronic obstructive pulmonary disease, *PAD* peripheral arterial disease, *CRP* C-reactive protein, *ΔPmean* mean aortic valve pressure gradient

#### Validation cohort for predictors of 1-year mortality identified in multiple regression analyses from bivariate comparisons and LASSO analyses

The multiple regression models for parameters from the bivariate comparisons (Table 3) and the LASSO analyses (Table 4), included patients with a documented EuroSCORE II (*n* = 1410). This patient cohort represents 79.5% of the patient cohort with available 1-year mortality data (*n* = 1774). To assess the stability and transferability of the model, the remaining 364 patients (20.5%) of this cohort with available 1-year mortality data but without documented EuroSCORE II were used as a validation cohort for the models shown in Tables 3 and 4. Missing data in the validation cohort were imputed. The AUC of the validation cohort (mean of the 20 AUCs across the imputed data sets) for the predictors identified by multiple regression analysis based on bivariate comparisons (Table 3) was 0.771 (95% CI 0.680–0.862). For the predictors identified by multiple regression based on LASSO analyses (Table 4), the AUC was 0.794 (95% CI: 0.699–0.888).

### Multivariable regression analysis of parameters from bivariate comparisons and LASSO-analysis between 30-day TAVR survivors and non-survivors

To identify predictors of 30-day mortality, bivariate comparisons were first performed with the variables used in the analyses for 1-year mortality (see Table 1: Demographics and comorbidities and Table 2: Pre-interventional examinations; data not shown). LASSO analyses for 30-day mortality were then performed using the same variables after multiple imputations. For comparison with EuroSCORE II, only patients with complete data sets and documented EuroSCORE II were included in these analyses (*n* = 1539).

In bivariate comparisons, six variables initially showed significant differences and were subsequently included in a multiple regression model for further analysis (Supplementary Table 3), with NYHA class IV being the only independent significant variable (OR 3.34, 95% CI 1.68–6.66, *p* = 0.001). In the LASSO analysis, NYHA class IV, age, chronic renal insufficiency, tricuspid valve disease (grade 2/3), sodium, and NT-proBNP before TAVR were included in at least 10 out of 20 models. However, in subsequent multiple regression analyses, only NYHA class IV (*p* < 0.001), chronic renal failure (*p* = 0.031) and pre-TAVR sodium (*p* = 0.035) were significant. The cut-off value determined by the Youden index was 139.5 mmol/l for sodium. For the combined model, the AUC for predicting 30-day mortality was 0.699 (95% CI 0.611–0.788), with the AUC for NYHA class IV alone being 0.623 (95% CI 0.551–0.701). In comparison, the AUC for 30-day mortality for EuroSCORE II is 0.680 (95% CI 0.604–0.756) and for the STS/ACC TAVR score is 0.7129 (95% CI 0.63323–0.79261). Additionally, we assessed the predictive performance of the 30-day mortality models using PPV and NPV. The combined 30-day mortality predictor model of this study achieved a PPV of 4.5% (95% CI 3.1–6.3%) and an NPV of 98.9% (95% CI 98.0–99.5%). For comparison, the EuroSCORE II model showed a PPV of 4.4% and a NPV of 99.4%, the STS/ACC TAVR score achieved a PPV of 4.4% (95% CI 3.1–6.0%) and an NPV of 99.4% (95% CI 98.5–99.8%).

### Evaluation of peri-interventional complications and their impact on 1-year and 30-day mortality

The most common peri-interventional complications during the TAVR-procedure were analyzed to assess their impact on 1-year and 30-day mortality. For the correlation of peri-interventional complications and 1-year mortality, the data of all patients with available information on survival status were used, analyzing both, the overall cohort (*n* = 1774) and a cohort that excluded patients who died within the first 30 days (*n* = 1726; Supplementary Table 4). In the overall cohort, only weak correlations were observed, with renal failure requiring renal replacement therapy (RRT) (phi = 0.21), extended post-interventional vasopressor therapy (phi = 0.19), pericardial tamponade (phi = 0.16), and cardiopulmonary resuscitation (phi = 0.15) showing the strongest relationships. After excluding patients who died within 30 days, major cerebral stroke (phi = 0.11) and length of hospital stay after the TAVR procedure (*r* = 0.11) showed the strongest correlations.

For 30-day mortality (Supplementary Table 5) the strongest correlations were observed with renal failure requiring RRT (phi = 0.32), vasopressor therapy for more than 6 h after TAVR (phi = 0.27), cardiopulmonary resuscitation (phi = 0.27), and pericardial tamponade (phi = 0.26).

## Discussion

Over the past two decades, recent studies have shown improved survival rates following TAVR procedures [13, 14]. This improvement is partly attributed to factors such as growing interventionist expertise, continuous advancements in prosthetic valve technology, and routine pre-procedural imaging, including screening CT [15]. This remarkable evolution in technical expertise and procedural skills has extended the use of TAVR beyond high-risk patients to intermediate and low-risk patients [1–3]. However, outcomes following TAVR are often limited, particularly in patients with specific pre-existing demographics and relevant comorbidities [16]. Thus, patient selection at the appropriate time and peri-procedural management remain significant challenges for clinicians. In this regard, the utility of commonly used surgical risk scores, such as EuroSCORE II, remains limited [17–19]. While most *PRE-TAVR* predictors are also included in the EuroSCORE II, its algorithm and factor weighting are primarily tailored to surgical patients undergoing coronary artery bypass grafting (CABG). As a result, first, while the EuroSCORE II is well established, it offers only moderate discrimination in predicting 30-day mortality in TAVR patients and tends to overestimate the risk of mortality [20, 21]. Second, the score is typically assessed well in advance of the procedure, introducing uncertainty when evaluating the patient’s clinical status in the pre-TAVR setting. Third, time-critical bedside use is challenging, as the EuroSCORE II requires the input of multiple parameters into a calculator or algorithm-based model before risk prediction can be applied. Furthermore, the EuroSCORE II includes a relatively large number of variables. As our study cohort lacks information on factors such as pulmonary hypertension, the EuroSCORE II could not be calculated retrospectively for all patients. Following the recognition of the limited performance of EuroSCORE II in TAVR, several modified risk scores have been developed and validated. These are, e.g., the PARTNER risk score, the FRANCE-2 score, the OBSERVANT risk score based on Italian registry data, the ACEF and ACEF-7 scores (which consider age, creatinine levels, and ejection fraction), the VARC-3 criteria and the American College of Cardiology TVT Registry risk model and STS/ACC TAVR score [10, 11, 22–26]. The STS/ACC TAVR score has recently been shown to be superior to other well-established risk scores, despite requiring only a small number of variables [12]. This is why we have also included it in the current study for comparison.

Despite the development of several modified risk scores, which in part already offer improved predictive accuracy compared to the EuroSCORE II, these tools have not been widely adopted in clinical practice. This limited implementation may be due to their inability to fully capture the complex and multifactorial nature of TAVR patients. As a result, there is an increasing recognition that traditional risk scores alone may not be sufficient for guiding clinical decision-making. Laurent et al. even suggest that simple bedside clinical assessments could provide more accurate predictions of outcomes after TAVR, emphasizing the importance of incorporating frailty and quality of life into the risk assessment process [27]. The underlying aspects of this analysis are entirely valid, as they emphasize the long-standing call for timely risk assessment and the need for a more comprehensive approach to the peri-procedural risk evaluation in TAVR [28–30].

The *PRE-TAVR* predictors identified in this study exhibited statistically significant prognostic superiority over EuroSCORE II, especially in predicting 1-year mortality. This may stem from their ability to consider a broad range of “real-time” parameters, reflecting the current state of the patient, including comorbidities and precisely pre-defined general clinical conditions in close temporal proximity to the TAVR procedure (e.g., NYHA IV, COPD Gold ≥ 2, atrial fibrillation, ΔPmean of the aortic valve ≥ 48.5 mmHg). Additionally, they account for dynamic laboratory markers, such as CRP and platelet count, which reflect the presence of outcome-relevant inflammation, infection, and bleeding risk. Although these factors can be partially addressed prior to intervention, our study does not directly demonstrate that such optimization improves outcomes. The optimal cut-off values identified by the model may reflect cohort-specific patterns and should therefore be interpreted cautiously until validated in independent datasets to ensure generalizability and to minimize the risk of overfitting.

Based on the large and comprehensive data set of the present study, the most significant independent predictor for 30-day mortality was NYHA class IV. This underscores the prognostic impact of pre-existing decompensated heart failure on outcomes and reinforces the importance of early diagnosis and timely decision-making for TAVR [31]. The evaluation of the role of early TAVR in asymptomatic patients with severe aortic stenosis is ongoing, yielding first promising results [4, 32]. Thus, in cases of clinically present decompensation or cardiogenic shock, alternative or staged management strategies should be considered. These may include balloon aortic valvuloplasty and/or Impella support followed by TAVR to optimize hemodynamic stability and transition the patient to a lower-risk profile for improved long-term outcomes been extended in recent years to [33–35].

The relevance of an optimized pre- and peri-procedural management of TAVR patients becomes even clearer when considering peri-procedural complications such as renal failure requiring RRT, the use of vasopressor therapy, cerebral stroke, and a prolonged in-hospital stay, which according to our data were also associated with increased 1-year mortality. Although these pathophysiological findings are well-established and known to correlate with poorer outcomes due to their prognostic value, partially addressed in previous studies, they highlight the complexity of patient profiles in TAVR. This underscores the need for timely pre-interventional assessments with the potential for early intervention of risk factors [16, 36–42].

With the ongoing demographic shift and the increasing complexity of patient populations, there is a growing overall need for more precise and personalized risk assessments in clinical practice. As the demand for efficient decision-making tools rises, there may be an opportunity to integrate predictors like *PRE-TAVR* into artificial intelligence (AI)-driven systems [43–45]. These tools can enable automated, real-time risk assessments, enhancing clinical decision-making and ensuring timely, individualized care without disrupting clinical workflows.

## Conclusions

This study highlights the importance of identifying and optimizing pre- and peri-procedural predictors, which may support improved risk stratification and management in TAVR patients, especially those with relevant comorbidities. Our findings show that PRE-TAVR predictors are more accurate than the EuroSCORE II and the STS/ACC TAVR score at predicting 1-year mortality. Unlike scoring systems, which often oversimplify complex clinical scenarios, the timely application of individualized *PRE-TAVR* predictors may support more precise patient management. In the future, integrating these validated predictors into clinical decision support systems could allow for more precise risk stratification and improve clinical decision-making.

### Limitations

Despite its comprehensive scope, the retrospective design of this study may introduce biases from data collection and patient selection. Due to the specific German healthcare context of the study and the specific heart valve systems used, the transferability of the results may be limited to similar healthcare systems and patient groups. The lack of external validation is an important limitation because it may restrict how generalizable our findings are to other patient populations. Although internal validation using an independent subset of our data produced consistent results, external validation in independent cohorts is necessary to confirm the robustness and broader applicability of our model. Another limitation is that the EuroSCORE II could not be calculated for the entire study population because some cases lacked the necessary variables for the retrospective data analysis. Consequently, direct comparisons between the PRE-TAVR predictors and the EuroSCORE II were limited to this group. This could lead to selection bias and limit the generalizability of our benchmarking results. Future studies should validate and refine these models for real-time clinical use. This requires consideration of the complex interplay of multiple clinical factors following TAVR to ensure robust performance in different patient populations.

## Supplementary Information

Below is the link to the electronic supplementary material.

ESM 1 (DOCX 33.7 KB)

## Data Availability

The data underlying this article will be shared on reasonable request to the corresponding author.

## References

[1] Van Mieghem NM, Deeb GM, Søndergaard L et al (2022) Self-expanding transcatheter vs surgical aortic valve replacement in intermediate-risk patients: 5-year outcomes of the SURTAVI randomized clinical trial. JAMA Cardiol 7(10):1000–1008. 10.1001/jamacardio.2022.2695 36001335 10.1001/jamacardio.2022.2695 PMC9403849

[2] Pibarot P, Ternacle J, Jaber WA et al (2020) Structural deterioration of transcatheter versus surgical aortic valve bioprostheses in the PARTNER-2 Trial. J Am Coll Cardiol 76(16):1830–1843. 10.1016/j.jacc.2020.08.049 33059828 10.1016/j.jacc.2020.08.049

[3] Mack MJ, Leon MB, Thourani VH et al (2023) Transcatheter aortic-valve replacement in low-risk patients at five years. N Engl J Med 389(21):1949–1960. 10.1056/NEJMoa2307447 37874020 10.1056/NEJMoa2307447

[4] Généreux P, Schwartz A, Oldemeyer B et al (2024) Design and rationale of the evaluation of transcatheter aortic valve replacement compared to surveillance for patients with asymptomatic severe aortic stenosis: The EARLY TAVR trial. Am Heart J 268:94–103. 10.1016/j.ahj.2023.11.019 38056546 10.1016/j.ahj.2023.11.019

[5] Mack MJ, Leon MB, Thourani VH et al (2019) Transcatheter aortic-valve replacement with a balloon-expandable valve in low-risk patients. N Engl J Med 380(18):1695–1705. 10.1056/NEJMoa1814052 30883058 10.1056/NEJMoa1814052

[6] Bhogal S, Rogers T, Aladin A et al (2023) TAVR in 2023: who should not get it? Am J Cardiol 193:1–18. 10.1016/j.amjcard.2023.01.040 36857839 10.1016/j.amjcard.2023.01.040

[7] Nashef SAM, Roques F, Sharples LD, et al (2012) EuroSCORE II. Eur J Cardiothorac Surg. discussion 41(4):734–744;744–745. 10.1093/ejcts/ezs043 10.1093/ejcts/ezs043 22378855

[8] Martin GP, Sperrin M, Ludman PF et al (2017) Inadequacy of existing clinical prediction models for predicting mortality after transcatheter aortic valve implantation. Am Heart J 184:97–105. 10.1016/j.ahj.2016.10.020 28224933 10.1016/j.ahj.2016.10.020 PMC5333927

[9] Halkin A, Steinvil A, Witberg G, et al (2016) Mortality prediction following transcatheter aortic valve replacement: a quantitative comparison of risk scores derived from populations treated with either surgical or percutaneous aortic valve replacement. The Israeli TAVR Registry Risk Model Accuracy Assessment (IRRMA) study. Int J Cardiol 215:227-231. 10.1016/j.ijcard.2016.04.038 10.1016/j.ijcard.2016.04.038 27128536

[10] Edwards FH, Cohen DJ, O’Brien SM et al (2016) Development and validation of a risk prediction model for in-hospital mortality after transcatheter aortic valve replacement. JAMA Cardiol 1(1):46–52. 10.1001/jamacardio.2015.0326 27437653 10.1001/jamacardio.2015.0326

[11] B I, C L, D H, et al. Predictive factors of early mortality after transcatheter aortic valve implantation: individual risk assessment using a simple score. Heart (British Cardiac Society). 2014;100(13). 10.1136/heartjnl-2013-305314 10.1136/heartjnl-2013-305314 24740804

[12] Arsalan M, Weferling M, Hecker F et al (2018) TAVI risk scoring using established versus new scoring systems: role of the new STS/ACC model. EuroIntervention 13(13):1520–1526. 10.4244/EIJ-D-17-00421 28994653 10.4244/EIJ-D-17-00421

[13] Leon MB, Smith CR, Mack M et al (2010) Transcatheter aortic-valve implantation for aortic stenosis in patients who cannot undergo surgery. N Engl J Med 363(17):1597–1607. 10.1056/NEJMoa1008232 20961243 10.1056/NEJMoa1008232

[14] Makkar RR, Thourani VH, Mack MJ et al (2020) Five-year outcomes of transcatheter or surgical aortic-valve replacement. N Engl J Med 382(9):799–809. 10.1056/NEJMoa1910555 31995682 10.1056/NEJMoa1910555

[15] Karra N, Sharon A, Massalha E et al (2024) Temporal trends in patient characteristics and clinical outcomes of TAVR: over a decade of practice. J Clin Med 13(17):5027. 10.3390/jcm13175027 39274239 10.3390/jcm13175027 PMC11396256

[16] Deutsch MA, Erlebach M, Burri M et al (2018) Beyond the five-year horizon: long-term outcome of high-risk and inoperable patients undergoing TAVR with first-generation devices. EuroIntervention 14(1):41–49. 10.4244/EIJ-D-17-00603 29581084 10.4244/EIJ-D-17-00603

[17] Biancari F, Juvonen T, Onorati F et al (2014) Meta-analysis on the performance of the EuroSCORE II and the Society of Thoracic Surgeons Scores in patients undergoing aortic valve replacement. J Cardiothorac Vasc Anesth 28(6):1533–1539. 10.1053/j.jvca.2014.03.014 25263775 10.1053/j.jvca.2014.03.014

[18] Wendt D, Thielmann M, Kahlert P et al (2014) Comparison between different risk scoring algorithms on isolated conventional or transcatheter aortic valve replacement. Ann Thorac Surg 97(3):796–802. 10.1016/j.athoracsur.2013.09.012 24594746 10.1016/j.athoracsur.2013.09.012

[19] Kuwaki K, Inaba H, Yamamoto T et al (2015) Performance of the EuroSCORE II and the Society of Thoracic Surgeons Score in patients undergoing aortic valve replacement for aortic stenosis. J Cardiovasc Surg (Torino) 56(3):455–462 25729918

[20] Watanabe Y, Hayashida K, Lefèvre T et al (2013) Is EuroSCORE II better than EuroSCORE in predicting mortality after transcatheter aortic valve implantation? Catheter Cardiovasc Interv 81(6):1053–1060. 10.1002/ccd.24702 23074135 10.1002/ccd.24702

[21] Durand E, Borz B, Godin M et al (2013) Performance analysis of EuroSCORE II compared to the original logistic EuroSCORE and STS scores for predicting 30-day mortality after transcatheter aortic valve replacement. Am J Cardiol 111(6):891–897. 10.1016/j.amjcard.2012.11.056 23337835 10.1016/j.amjcard.2012.11.056

[22] Capodanno D, Barbanti M, Tamburino C et al (2014) A simple risk tool (the OBSERVANT score) for prediction of 30-day mortality after transcatheter aortic valve replacement. Am J Cardiol 113(11):1851–1858. 10.1016/j.amjcard.2014.03.014 24837264 10.1016/j.amjcard.2014.03.014

[23] Ranucci M, Castelvecchio S, Conte M et al (2011) The easier, the better: age, creatinine, ejection fraction score for operative mortality risk stratification in a series of 29,659 patients undergoing elective cardiac surgery. J Thorac Cardiovasc Surg 142(3):581–586. 10.1016/j.jtcvs.2010.11.064 21703638 10.1016/j.jtcvs.2010.11.064

[24] Alnajar A, Chatterjee S, Chou BP et al (2021) Current surgical risk scores overestimate risk in minimally invasive aortic valve replacement. Innovations (Phila) 16(1):43–51. 10.1177/1556984520971775 33269957 10.1177/1556984520971775

[25] Denegri A, Mehran R, Holy E et al (2019) Post procedural risk assessment in patients undergoing trans aortic valve implantation according to the age, creatinine, and ejection fraction-7 score: advantages of age, creatinine, and ejection fraction-7 in stratification of post-procedural outcome. Catheter Cardiovasc Interv 93(1):141–148. 10.1002/ccd.27806 30269398 10.1002/ccd.27806

[26] VARC-3 WRITING COMMITTEE:, Généreux P, Piazza N, et al (2021) Valve Academic Research Consortium 3: updated endpoint definitions for aortic valve clinical research. J Am Coll Cardiol. 77(21):2717–2746. 10.1016/j.jacc.2021.02.038 10.1016/j.jacc.2021.02.038 33888385

[27] Laurent M, Fournet M, Feit B et al (2013) Simple bedside clinical evaluation versus established scores in the estimation of operative risk in valve replacement for severe aortic stenosis. Arch Cardiovasc Dis 106(12):651–660. 10.1016/j.acvd.2013.09.001 24231053 10.1016/j.acvd.2013.09.001

[28] Afilalo J, Lauck S, Kim DH et al (2017) Frailty in older adults undergoing aortic valve replacement: the FRAILTY-AVR study. J Am Coll Cardiol 70(6):689–700. 10.1016/j.jacc.2017.06.024 28693934 10.1016/j.jacc.2017.06.024

[29] Kleczynski P, Dziewierz A, Bagienski M et al (2017) Impact of frailty on mortality after transcatheter aortic valve implantation. Am Heart J 185:52–58. 10.1016/j.ahj.2016.12.005 28267475 10.1016/j.ahj.2016.12.005

[30] Dautzenberg L, van Aarle TTM, Stella PR, Emmelot-Vonk M, Weterman MA, Koek HL (2022) The impact of frailty on adverse outcomes after transcatheter aortic valve replacement in older adults: a retrospective cohort study. Catheter Cardiovasc Interv 100(3):439–448. 10.1002/ccd.30320 35830708 10.1002/ccd.30320 PMC9545405

[31] Parikh PB, Mack M, Stone GW et al (2024) Transcatheter aortic valve replacement in heart failure. Eur J Heart Fail 26(2):460–470. 10.1002/ejhf.3151 38297972 10.1002/ejhf.3151

[32] Généreux P, Schwartz A, Oldemeyer JB, et al (2024) Transcatheter aortic-valve replacement for asymptomatic severe aortic stenosis. N Engl J Med. Published online. 10.1056/NEJMoa2405880 10.1056/NEJMoa2405880 39466903

[33] Jabri A, Ayyad M, Albandak M, et al (2024) Outcomes following TAVR in patients with cardiogenic shock: a systematic review and meta-analysis. Cardiovasc Revasc Med. Published online. S1553–8389(24)00622–5. 10.1016/j.carrev.2024.08.002 10.1016/j.carrev.2024.08.002 39209579

[34] Singh V, Mendirichaga R, Inglessis-Azuaje I, Palacios IF, O’Neill WW (2018) The role of impella for hemodynamic support in patients with aortic stenosis. Curr Treat Options Cardiovasc Med 20(6):44. 10.1007/s11936-018-0644-9 29682687 10.1007/s11936-018-0644-9

[35] Almajed MR, Mahmood S, Obri M et al (2023) Application of impella mechanical circulatory support devices in transcatheter aortic valve replacement and balloon aortic valvuloplasty: a single-center experience. Cardiovasc Revasc Med 53:1–7. 10.1016/j.carrev.2023.03.006 37012106 10.1016/j.carrev.2023.03.006

[36] Gupta T, Goel K, Kolte D et al (2017) Association of chronic kidney disease with in-hospital outcomes of transcatheter aortic valve replacement. JACC Cardiovasc Interv 10(20):2050–2060. 10.1016/j.jcin.2017.07.044 29050621 10.1016/j.jcin.2017.07.044

[37] Iskander M, Jamil Y, Forrest JK et al (2023) Cerebral embolic protection in transcatheter aortic valve replacement. Struct Heart 7(4):100169. 10.1016/j.shj.2023.100169 37520138 10.1016/j.shj.2023.100169 PMC10382985

[38] Dvir D, Généreux P, Barbash IM et al (2014) Acquired thrombocytopenia after transcatheter aortic valve replacement: clinical correlates and association with outcomes. Eur Heart J 35(38):2663–2671. 10.1093/eurheartj/ehu082 24598983 10.1093/eurheartj/ehu082

[39] Ashley KE, Hillegass WB (2019) Clopidogrel pretreatment may reduce early acquired thrombocytopenia after transcatheter aortic valve replacement (TAVR). Catheter Cardiovasc Interv 94(6):818–819. 10.1002/ccd.28569 31737991 10.1002/ccd.28569

[40] Zhang K, Troeger W, Kuhn M et al (2022) Evaluation of systemic inflammation in response to remote ischemic preconditioning in patients undergoing transcatheter aortic valve replacement (TAVR). Rev Cardiovasc Med 23(1):20. 10.31083/j.rcm2301020 35092212 10.31083/j.rcm2301020

[41] Puehler T, Pommert NS, Freitag-Wolf S et al (2024) Tricuspid regurgitation and TAVR: outcomes, risk factors and biomarkers. J Clin Med 13(5):1474. 10.3390/jcm13051474 38592316 10.3390/jcm13051474 PMC10934629

[42] Lauridsen MD, Valentin JB, Strange JE et al (2023) Mortality in patients with chronic obstructive pulmonary disorder undergoing transcatheter aortic valve replacement: the importance of chronic obstructive pulmonary disease exacerbation. Am Heart J 262:100–109. 10.1016/j.ahj.2023.04.016 37116603 10.1016/j.ahj.2023.04.016

[43] Castaneda C, Nalley K, Mannion C et al (2015) Clinical decision support systems for improving diagnostic accuracy and achieving precision medicine. J Clin Bioinforma 5:4. 10.1186/s13336-015-0019-3 25834725 10.1186/s13336-015-0019-3 PMC4381462

[44] Rambach T, Gleim P, Mandelartz S, Heizmann C, Kunze C, Kellmeyer P (2024) Challenges and facilitation approaches for the participatory design of AI-based clinical decision support systems: protocol for a scoping review. JMIR Res Protoc 13:e58185. 10.2196/58185 39235846 10.2196/58185 PMC11413541

[45] Lu Y, Melnick ER, Krumholz HM (2022) Clinical decision support in cardiovascular medicine. BMJ 377:e059818. 10.1136/bmj-2020-059818 35613721 10.1136/bmj-2020-059818

