# Supplementary material for: Patient risk evaluation for transcatheter aortic valve replacement (PRE-TAVR) — identification of real-time predictors of short- and long-term mortality
Source: Clin Res Cardiol. 2025 Jul 7;114(11):1574–84. doi: 10.1007/s00392-025-02704-6 (PMC12540637; doi:10.1007/s00392-025-02704-6)
Supplement: Supplementary file 1 — (DOCX 33.7 KB) [file 392_2025_2704_MOESM1_ESM.docx]

**Appendix**

**Supplement Table 1. Multivariable regression analysis on significant parameters identified from the bivariate analysis, comparing 1-year survivors and non-survivors** **(Table 1: demographics and comorbidities and Table 2: pre-interventional examinations).** The dependent variable was “survived”. Abbreviations: CHD: coronary heart disease; NYHA: New York Heart Association; KDIGO: Kidney Disease Improve Global Outcomes; COPD: chronic obstructive pulmonary disease; PAD: peripheral arterial disease; CRP: C-reactive protein; NT-proBNP: N-terminal prohormone of brain natriuretic peptide; Vmax: peak transvalvular velocity of aortic valve; ΔPmean: mean aortic valve pressure gradient.

|  | **OR (95% CI)** | **p-value** |
| --- | --- | --- |
| **Demographics and comorbidities** |  |  |
| Age (years) | 1.08 (1.03-1.12) | **<0.001** |
| CHD | 1.15 (0.73-1.83) | 0.548 |
| Heart failure NYHA III | 1.38 (0.76-2.51) | 0.284 |
| Heart failure NYHA IV | 3.11 (1.55-6.24) | **0.001** |
| Atrial fibrillation | 1.60 (0.99-2.59) | 0.058 |
| Chronic renal failure  KDIGO ≥ stage 3 | 1.14 (0.71-1.85) | 0.588 |
| COPD ≥ GOLD 2 | 2.31 (1.31-4.08) | **0.004** |
| Cerebral stroke | 3.32 (1.26-8.75) | **0.016** |
| Malignant disease | 1.93 (0.90-4.14) | 0.091 |
| PAD (≥ stage 2) | 1.58 (0.79-3.15) | 0.199 |
| **Pre-interventional examinations** |  |  |
| Pleural effusion (chest X-ray) | 0.71 (0.37-1.37) | 0.309 |
| Congestion signs (chest X-ray) | 1.15 (0.47-2.84) | 0.761 |
| CRP (mg/l) | 1.20 (1.00-1.43) | **0.048** |
| Hemoglobin (g/L) | 1.00 (0.97-1.02) | 0.802 |
| Hematocrit (L/L) | 2.75 (0.00-55306) | 0.842 |
| Sodium (mmol/L) | 0.96 (0.90-1.01) | 0.134 |
| NT proBNP (pg/mL) | 1.15 (0.94-1.42) | 0.171 |
| Vmax (mmHg) | 1.90 (0.83-4.34) | 0.131 |
| ΔPmean (mmHg) | 0.96 (0.92-0.99) | **0.025** |
| Vitium (grade 2/3) of the tricuspid valve | 1.30 (0.79-2.15) | 0.304 |

**Supplement Table 2. Multivariable Regression Analysis on Imputed Data.** Parameters from Table 1 (demographics and comorbidities) and Table 2 (pre-interventional examinations) appearing in more than 10 LASSO Models, comparing 1-year survivors and non-survivors, with “survived” as the dependent variable. Abbreviations: BMI: body mass index; CHD: coronary heart disease; NYHA: New York Heart Association; KDIGO: Kidney Disease Improve Global Outcomes; COPD: chronic obstructive pulmonary disease; PAD: peripheral arterial disease; CRP: C-reactive protein; NT-proBNP: N-terminal prohormone of brain natriuretic peptide; LVEF: left ventricular ejection fraction; ΔPmean: mean aortic valve pressure gradient.

|  | **OR (95% CI)** | **p-value** |
| --- | --- | --- |
| **Demographics and comorbidities** |  |  |
| Age (years) | 1.06 (1.02-1.10) | **0.001** |
| Male sex | 0.72 (0.48-1.09) | 0.122 |
| BMI | 0.99 (0.95-1.03) | 0.509 |
| CHD | 1.25 (0.86-1.82) | 0.251 |
| Heart failure NYHA III | 1.37 (0.86-2.19) | 0.188 |
| Heart failure NYHA IV | 2.50 (1.41-4.44) | **0.002** |
| Diabetes mellitus | 0.84 (0.57-1.22) | 0.360 |
| Chronic renal failure  KDIGO ≥ stage 3 | 1.65 (1.11-2.44) | **0.012** |
| Renal replacement therapy | 0.56 (0.15-2.08) | 0.384 |
| COPD ≥ GOLD 2 | 2.07 (1.28-3.34) | **0.003** |
| Apoplexy | 3.02 (1.40-6.50) | **0.005** |
| Malignant disease | 2.07 (1.16-3.70) | **0.014** |
| PAD (≥ stage 2) | 1.57 (0.89-2.75) | 0.116 |
| Pacemaker | 0.39 (0.21-0.74) | **0.004** |
| Atrial fibrillation | 1.43 (0.97-2.11) | 0.075 |
| **Pre-interventional examinations** |  |  |
| 1st degree AV block | 0.71 (0.36-1.40) | 0.316 |
| CRP (mg/l) | 1.13 (0.98-1.31) | 0.095 |
| Hemoglobin (g/L) | 0.99 (0.98-1.00) | 0.099 |
| Platelet count (g/L) | 0.99 (0.99-1.00) | **0.002** |
| Sodium (mmol/L) | 0.95 (0.90-0.99) | **0.014** |
| NT proBNP (pg/mL) | 1.25 (0.99-1.56) | 0.054 |
| Diameter of ascending aorta | 1.00 (0.95-1.05) | 0.919 |
| LVEF ≤45% | 0.63 (0.39-1.02) | 0.059 |
| ΔPmean (mmHg) | 0.98 (0.97-0.99) | **0.004** |
| Vitium (grade 2/3) of the mitral valve | 0.83 (0.55-1.26) | 0.383 |
| Vitium (grade 2/3) of the tricuspid valve | 1.45 (0.96-2.20) | 0.081 |

**Supplement Table 3** **Multivariable regression analysis on significant parameters identified from the bivariate analysis, comparing 30-day survivors and non-survivors (Table 1: demographics and comorbidities and Table 2: pre-interventional examinations). The dependent variable was “survived”.** Abbreviations: NYHA: New York Heart Association; NT proBNP: N-terminal prohormone of brain natriuretic peptide.

|  | **OR (95% CI)** | **p-value** |
| --- | --- | --- |
| Age (years) | 1.03 (0.98-1.09) | 0.200 |
| Heart failure NYHA IV | 3.34 (1.68-6.66) | **0.001** |
| Chronic renal failure  KDIGO ≥ stage 3 | 1.49 (0.75-2.98) | 0.259 |
| Atrial fibrillation | 0.86 (0.43-1.69) | 0.659 |
| NT proBNP (pg/mL) | 1.20 (0.93-1.54) | 0.154 |
| Vitium (grade 2/3) of the tricuspid valve | 1.48 (0.72-3.07) | 0.290 |

**Supplement Table 4. Peri-interventional complications and their impact on 1-year mortality.** Abbreviations: MI: myocardial infarction; SAVR: surgical aortic valve replacement; RRT: renal replacement therapy. Phi/r from 0.1: weak correlation, from 0.3: medium correlation, from 0.5: strong correlation. *: excluding 30-day non-survivors. ^#^: r according to the Mann-Whitney test. ^1^: n (%); ^3^: Median (IQR).

|  | **n=** | **1-year**  **survivors** | **1-year**  **non-survivors** | **phi/r** | **phi/r*** |
| --- | --- | --- | --- | --- | --- |
| Cardiopulmonary resuscitation^1^ | 1774 | 13 (0.8) | 11 (7.0) | 0.15 | 0.03 |
| Dissection of the aorta^1^ | 1774 | 4 (0.2) | 1 (0.6) | 0.02 | 0.01 |
| Acute coronary intervention for periinterventional MI^1^ | 1774 | 1 (0.1) | 1 (0.6) | 0.01 | 0.01 |
| Switch to SAVR^1^ | 1774 | 3 (0.2) | 3 (1.9) | 0.08 | 0.01 |
| Catecholamine therapy > 6 hours postintervention^1^ | 1774 | 30 (1.9) | 20 (12.7) | 0.19 | 0.05 |
| New onset atrial fibrillation^1^ | 1774 | 30 (1.9) | 6 (3.8) | 0.04 | 0.06 |
| Pacemaker implantation^1^ | 1771 | 203 (12.6) | 30 (19.4) | 0.06 | 0.06 |
| Pericardial tamponade^1^ | 1774 | 34 (2.1) | 18 (11.4) | 0.16 | 0.01 |
| Renal failure requiring RRT^1^ | 1774 | 5 (0.3) | 12 (7.6) | 0.21 | 0.02 |
| Dissection of the femoral artery^1^ | 1774 | 14 (0.9) | 3 (1.9) | 0.03 | 0.02 |
| Failure of the occlusion system of the arterial access route^1^ | 1774 | 31 (1.9) | 4 (2.5%) | 0.01 | 0.002 |
| Vascular operation^1^ | 1774 | 67 (4.1) | 19 (12.0) | 0.10 | 0.05 |
| Transfusion within the first 48 hours after TAVR^1^ | 1774 | 82 (5.1) | 22 (13.9) | 0.11 | 0.08 |
| Minor Stroke^1^ | 1774 | 9 (0.6) | 7 (4.4) | 0.01 | 0.02 |
| Major Stroke^1^ | 1774 | 29 (1.8) | 2 (0.9) | 0.12 | 0.11 |
| Duration of stay (days) after TAVR^3^ | 1733 | 7.0 (5.0-10.0) | 10.0 (6.0-14.0) | 0.12^#^ | 0.11^#^ |

**Supplement Table 5. Peri-interventional complications and their impact on 30-day mortality.** Abbreviations: MI: myocardial infarction; SAVR: surgical aortic valve replacement; RRT: renal replacement therapy. Phi/r from 0.1: weak correlation, from 0.3: medium correlation, from 0.5: strong correlation. ^#^: r according to the Mann-Whitney test. ^1^: n (%); ^3^: Median (IQR).

|  | **n=** | **30-day**  **survivors** | **30-day**  **non-survivors** | **phi/r** |
| --- | --- | --- | --- | --- |
| Cardiopulmonary resuscitation^1^ | 2255 | 32 (1.5) | 17 (24.6) | 0.27 |
| Dissection of the aorta^1^ | 2256 | 4 (0.2) | 2 (2.9) | 0.09 |
| Acute coronary intervention for peri-interventional MI^1^ | 2256 | 16 (0.7) | 3 (4.3) | 0.07 |
| Switch to SAVR^1^ | 2256 | 3 (0.1) | 3 (4.3) | 0.14 |
| Catecholamine therapy > 6 hours postintervention^1^ | 2255 | 92 (4.2) | 27 (39.1) | 0.27 |
| New onset atrial fibrillation^1^ | 2255 | 62 (28) | 2 (2.9) | 0.001 |
| Pacemaker implantation^1^ | 2256 | 314 (14.4) | 11 (16.7) | 0.01 |
| Pericardial tamponade^1^ | 2256 | 38 (1.7) | 17 (24.6) | 0.26 |
| Renal failure requiring RRT^1^ | 2256 | 31 (1.4) | 20 (29.0) | 0.32 |
| Dissection of the femoral artery^1^ | 2256 | 18 (0.8) | 2 (2.9) | 0.04 |
| Failure of the occlusion system of the arterial access route^1^ | 2256 | 54 (2.5) | 4 (5.8) | 0.04 |
| Vascular operation^1^ | 2256 | 93 (4.3) | 13 (18.8) | 0.12 |
| Transfusion within the first 48 hours after TAVR^1^ | 2256 | 145 (6.6) | 16 (23.2) | 0.11 |
| Minor Stroke^1^ | 2256 | 17 (0.8) | 2 (2.9) | 0.001 |
| Major Stroke^1^ | 2256 | 30 (1.4) | 1 (1.4) | 0.04 |
| Duration of stay (days) after TAVR^3^ | 2194 | 7.0 (5.0-10.0) | 10.5 (8.25-12.0) | 0.04^#^ |
